# Supplementary material for: Full-Length Transcriptome Sequencing-Based Analysis of Pinus sylvestris var. mongolica in Response to Sirex noctilio Venom
Source: Insects. 2022 Mar 30;13(4):338. doi: 10.3390/insects13040338 (PMC9029201; doi:10.3390/insects13040338)
Supplement: Supplementary file 1 [file insects-13-00338-s001.zip › Table S1.pdf]

Table S1. The quality assessment of the 27 samples.

Sample	Concentration (ng/μL)	Total quality (μg)	OD260/280	RIN
CK1_0h_	555.00	16.65	2.11	8.30
CK2_0h	663.5	23.22	2.09	8.30
CK3_0h	915.30	32.04	2.06	8.30
CK1_24h	819.90	22.96	2.11	7.30
CK2_24h	765.50	21.43	2.17	7.90
CK3_24h	696.80	19.51	2.08	6.70
CK1_72h	707.70	24.77	2.10	7.70
CK2_72h	766.30	19.16	2.05	7.90
CK3_72h	861.80	30.16	2.06	7.80
PI1_0h	1087.20	21.74	2.10	8.10
PI2_0h	857.50	17.15	2.16	8.10
PI3_0h	1039.30	20.79	2.13	8.00
PI1_24h	601.60	18.05	2.07	8.10
PI2_24h	642.10	19.26	2.09	8.20
PI3_24h	833.00	23.32	2.10	7.90
PI1_72h	836.10	20.90	2.07	8.30
PI2_72h	803.50	20.09	2.05	8.40
PI3_72h	798.10	19.95	2.07	8.20
PW_0h	424.20	8.48	2.03	7.60
PW_0h	300.90	6.02	2.08	8.00
PW_0h	328.20	6.56	2.09	8.10
PW_24h	1102.20	30.86	2.12	7.50
PW_24h	472.80	13.24	2.02	8.00
PW_24h	638.70	19.16	2.10	7.40
PW_72h	741.40	25.95	2.08	8.30
PW_72h	534.30	18.70	1.98	8.30
PW_72h	937.20	32.80	2.05	8.40
