# Supplementary material for: Full-Length Transcriptome Sequencing-Based Analysis of Pinus sylvestris var. mongolica in Response to Sirex noctilio Venom
Source: Insects. 2022 Mar 30;13(4):338. doi: 10.3390/insects13040338 (PMC9029201; doi:10.3390/insects13040338)
Supplement: Supplementary file 1 [file insects-13-00338-s001.zip › Table S2.pdf]

Table S2. Summary of RNA sequencing data from 27 RNA libraries and comparison with full-length unigenes.

Sample	Clean reads	Mapped reads	Mapped ratio
CK1_0h_	52048226	42655426	81.95%
CK2_0h	43494680	36396150	83.68%
CK3_0h	47011562	38802952	82.54%
CK1_24h	48012518	39414898	82.09%
CK2_24h	52849158	42905310	81.18%
CK3_24h	47053344	39369962	83.67%
CK1_72h	49928134	41076888	82.27%
CK2_72h	54501374	44969844	82.51%
CK3_72h	44980644	38276898	85.10%
PI1_0h	51511470	43028316	83.53%
PI2_0h	45743680	36931666	80.74%
PI3_0h	47695470	39060402	81.90%
PI1_24h	43949758	36986670	84.16%
PI2_24h	48239756	40078062	83.08%
PI3_24h	50087732	40675468	81.21%
PI1_72h	47275746	39077014	82.66%
PI2_72h	45877602	37697492	82.17%
PI3_72h	47705738	39345140	82.47%
PW_0h	46567316	39231330	84.25%
PW_0h	44760406	36931568	82.51%
PW_0h	46431932	38805482	83.57%
PW_24h	46565662	39018804	83.79%
PW_24h	44655040	36934664	82.71%
PW_24h	45966012	38118612	82.93%
PW_72h	47105604	38097180	80.88%
PW_72h	48583032	39044078	80.37%
PW_72h	52043576	43108640	82.83%
