# Supplementary figures and images for: Full-Length Transcriptome Sequencing-Based Analysis of Pinus sylvestris var. mongolica in Response to Sirex noctilio Venom
Source: Insects. 2022 Mar 30;13(4):338. doi: 10.3390/insects13040338 (PMC9029201; doi:10.3390/insects13040338)

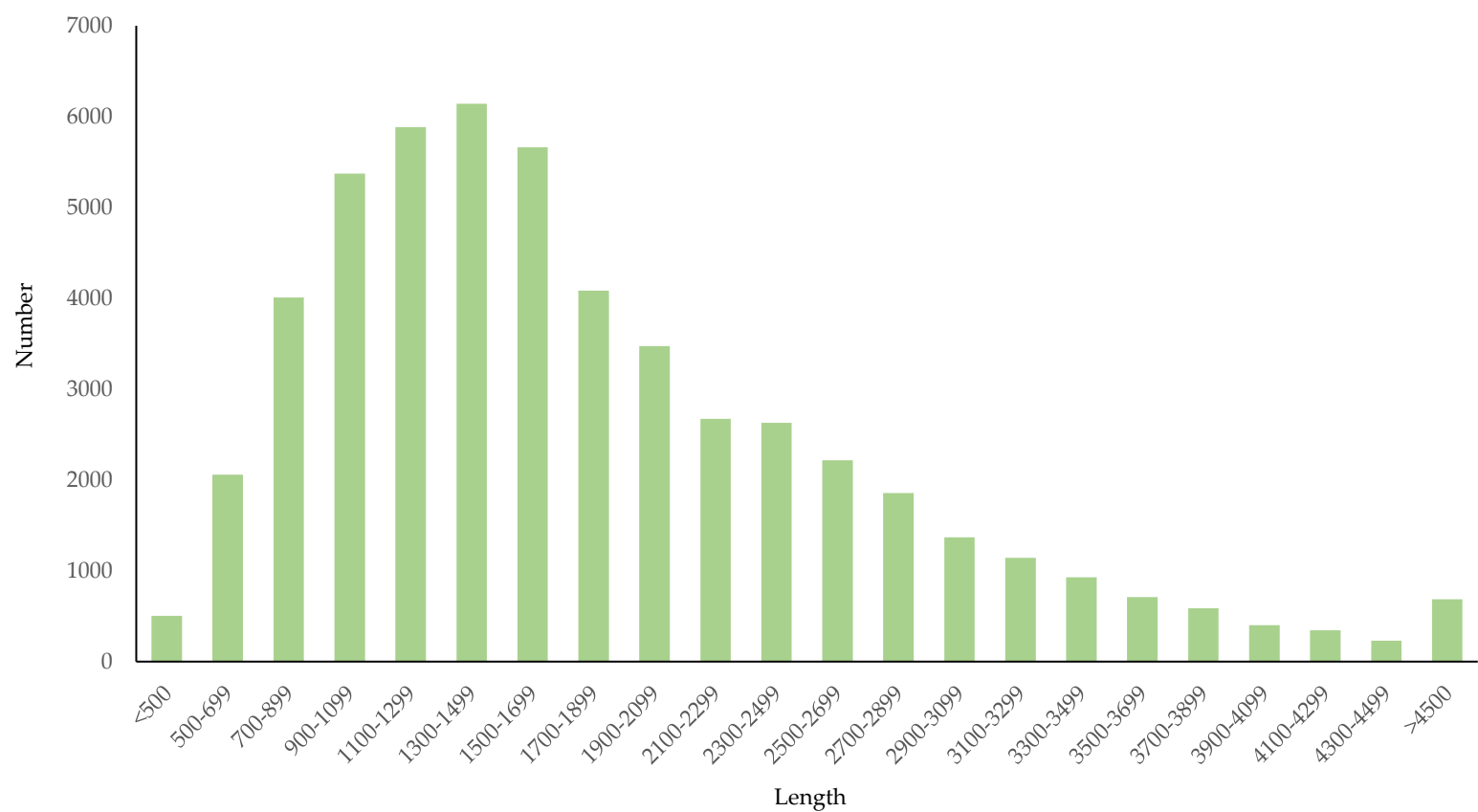

**Figure S1.** The length distribution of unigenes

Supplement: Supplementary file 1 [file insects-13-00338-s001.zip › Figure S1.pdf]

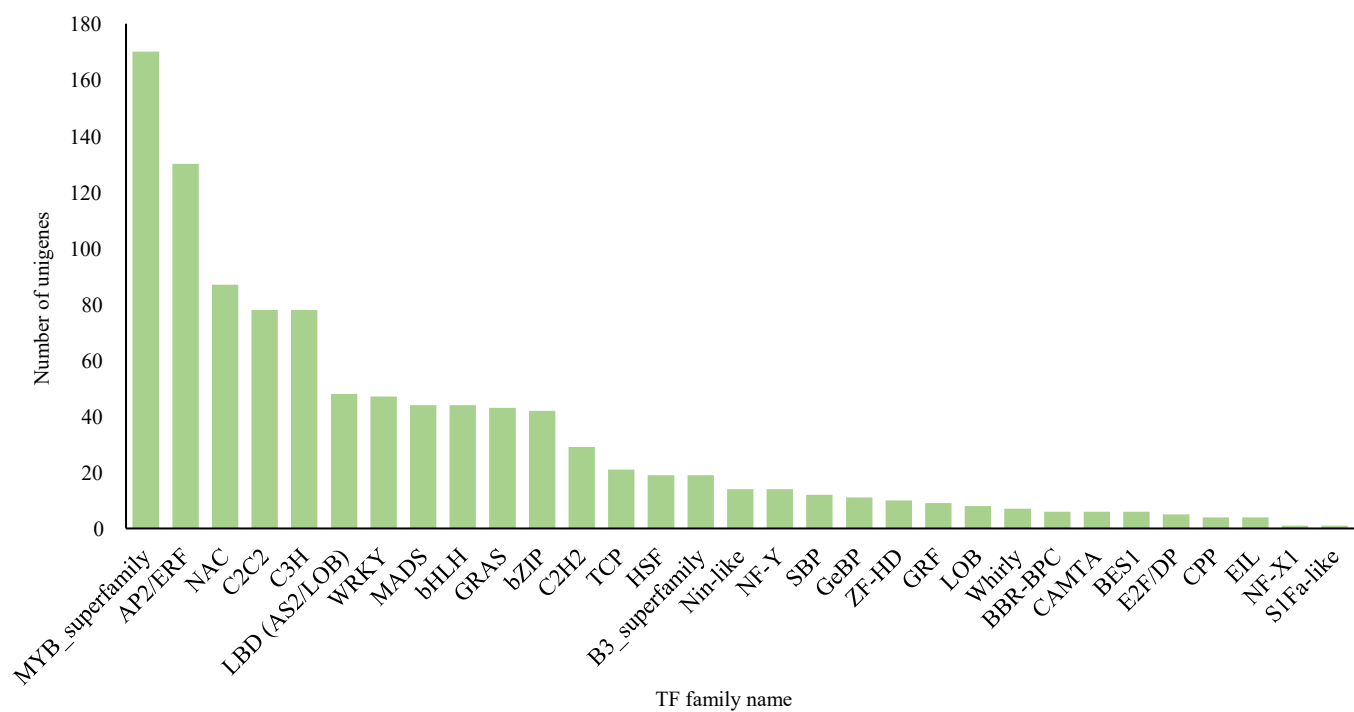

**Figure S2.** Unigene number distribution of TF families

Supplement: Supplementary file 1 [file insects-13-00338-s001.zip › Figure S2.pdf]
